# Ticagrelor monotherapy after a short course of dual antiplatelet therapy with ticagrelor plus aspirin following percutaneous coronary intervention in patients with versus without diabetes mellitus: a meta-analysis of randomized trials

**DOI:** 10.1186/s12872-024-03836-9

**Published:** 2024-03-19

**Authors:** Chen Ning, Fang Ling, Deyi Liu, Zhang Zhi

**Affiliations:** 1 Department of Cardiology, Shandong Tai An 88 Hospital, Shandong Tai An, 271000 People’s Republic of China; 2 Department of Cardiology, The First College of Clinical Medical Science, China Three Gorges University, Yichang Central People’s Hospital, Hubei Yichang, 443000 People’s Republic of China

**Keywords:** Ticagrelor monotherapy, Dual antiplatelet therapy, Diabetes mellitus, Cardiovascular events, Bleeding risk, Percutaneous coronary intervention, Major adverse cardiac events, Thrombolysis in myocardial infarction, Bleeding defined by the Academic Research Consortium

## Abstract

**Background:**

Cardiovascular disease (CVD) is one among the major causes of mortality all round the globe. Several anti-platelet regimens have been proposed following percutaneous coronary intervention (PCI). In this analysis, we aimed to show the adverse clinical outcomes associated with ticagrelor monotherapy after a short course of dual antiplatelet therapy (DAPT) with ticagrelor and aspirin following PCI in patients with versus without diabetes mellitus (DM).

**Methods:**

Electronic databases were searched by four authors from September to November 2023. Cardiovascular outcomes and bleeding events were the endpoints of this analysis. Revman 5.4 software was used to conduct this meta-analysis. Risk ratio (RR) and 95% confidence intervals (CI) were used to represent the results which were generated.

**Results:**

Three studies with a total number of 22,574 participants enrolled from years 2013 to 2019 were included in this analysis. Results of this analysis showed that DM was associated with significantly higher risks of major adverse cardiovascular events (RR: 1.73, 95% CI: 1.49 – 2.00; *P* = 0.00001), all-cause mortality (RR: 2.15, 95% CI: 1.73 – 2.66; *P* = 0.00001), cardiac death (RR: 2.82, 95% CI: 1.42 – 5.60; *P* = 0.003), stroke (RR: 1.78, 95% CI: 1.16 – 2.74; *P* = 0.009), myocardial infarction (RR: 1.63, 95% CI: 1.17 – 2.26; *P* = 0.004) and stent thrombosis (RR: 1.74, 95% CI: 1.03 – 2.94; *P* = 0.04) when compared to patients without DM.

However, thrombolysis in myocardial infarction (TIMI) defined minor and major bleedings, bleeding defined according to the academic research consortium (BARC) type 3c (RR: 1.31, 95% CI: 0.14 – 11.90; *P* = 0.81) and BARC type 2, 3 or 5 (RR: 1.17, 95% CI: 0.85 – 1.62; *P* = 0.34) were not significantly different.

**Conclusion:**

In patients who were treated with ticagrelor monotherapy after a short course of DAPT with ticagrelor and aspirin, DM was an independent risk factor for the significantly increased adverse cardiovascular outcomes. However, TIMI and BARC defined bleeding events were not significantly different in patients with versus without DM.

## Background

Cardiovascular disease (CVD) is one among the major causes of mortality all round the globe [1]. Several anti-platelet regimens have been proposed following percutaneous coronary intervention (PCI) [2]. Since platelet hyperactivity is more common with diabetes mellitus (DM) [3] and patients with DM are often less responsive to aspirin and clopidogrel [4], newer anti-platelet regimens with novel potent anti-platelet agents have been suggested [5] to prevent thrombosis in the DM population.

For years, oral dual antiplatelet therapy (DAPT) consisting of Aspirin and clopidogrel has been used as the ideal anti-thrombotic therapy for patients with CVD and those undergoing PCI [6]. However, higher rates of cardiovascular events keep rising especially among patients with DM prompting for the identification of novel and alternative anti-platelet regimens to optimize platelet inhibition [7]. Even though a prolonged duration of DAPT use in patients with DM was considered, no statistically significant benefit of longer DAPT use was observed in patients with DM [8].

Ticagrelor, a potent anti-platelet agent recently showed favorable results following revascularization in the general population with CVD [9]. However, longterm ticagrelor with aspirin as a DAPT regimen has shown to be associated with bleeding events [10].

Nevertheless, ticagrelor monotherapy after a short course of DAPT with ticagrelor and aspirin has shown promising results in the general population with CVD. The Ticagrelor Monotherapy after 3 Months in the Patients treated with New generation Sirolimus Eluting Stent for Acute Coronary Syndrome (TICO) trial compared ticagrelor monotherapy after 3-month DAPT use versus ticagrelor based 12-month DAPT use in patients with high ischemic risk acute coronary syndrome and the results showed both treatment regimens to be equally effective among the 3056 patients with high ischemic risk [11]. A meta-analysis published in the year 2022 showed ticagrelor monotherapy after a short term DAPT use of 1–3 months to significantly reduce mortality and bleeding risks without causing any increase in stent thrombosis or myocardial infarction [12].

In this analysis, we aimed to show the adverse clinical outcomes associated with ticagrelor monotherapy after a short course of DAPT with ticagrelor and aspirin following PCI in patients with versus without DM.

## Methods

### Search databases and the search process

Publications were searched from common electronic databases (from September 2023 till November 2023). The databases included Excerpta Medica Database (EMBASE), Medical Literature Analysis and Retrieval System Online (MEDLINE), Google Scholar, Web of Science, http://www.Clinicaltrials.gov, and the Cochrane database. Publications based on ticagrelor monotherapy after a short course of DAPT with ticagrelor and aspirin in patients with versus without DM were considered relevant.

To proceed with the search of publications, the following search terms or phrases were used:

- “Ticagrelor monotherapy and diabetes mellitus and/or percutaneous coronary intervention”;
- “Dual antiplatelet therapy with ticagrelor and aspirin in diabetes mellitus”;
- “Ticagrelor monotherapy and percutaneous coronary intervention”;
- “percutaneous coronary intervention and novel antiplaltelet agents”;
- “percutaneous coronary intervention and dual antiplatelet therapy”;
- “percutaneous coronary intervention and monotherapy”;
- “acute coronary syndrome and dual antiplatelet therapy”;
- “acute coronary syndrome and monotherapy”;
- “acute coronary syndrome and ticagrelor and diabetes mellitus”.

Abbreviations like “PCI”, “DAPT” and “ACS” were used during this search procedure.

The above mentioned terms or phrases were searched from respective databases and the outcomes were carefully assessed prior to selection of studies.

### Inclusion and exclusion criteria

The inclusion criteria were:

- Randomized trials based on the comparison of ticagrelor monotherapy after a short duration of DAPT with ticagrelor and aspirin in patients with versus without DM;
- Study researches that reported adverse clinical outcomes and/or bleeding events among their endpoints.

The exclusion criteria were:

- Studies that were non-randomized trials or studies that were not derived from randomized trials, research that were observational studies, systematic reviews and meta-analyses, and literature reviews;
- Studies that were case studies or editorials;
- Studies that did not report adverse clinical or bleeding outcomes;
- Studies that were repetitive or simply repeated studies derived from same trials or cohort studies.

### Outcomes, duration of anti-platelet therapy and follow-up duration

The outcomes which were reported in the original studies have been listed in Table 1.

**Table 1:** Endpoints reported in the original studies

Studies	Endpoints	Duration of DAPT and monotherapy	Follow up time period
**Global Leaders **[13]	All-cause mortality, MI, stroke, any revascularization, TVR, definite stent thrombosis, MACE, POCE, NACE, BARC 3c, BARC 2, 3 or 5 bleeding	1 month DAPT followed by ticagrelor monotherapy	24 months
**Twilight **[14]	Death, MI or stroke, cardiovascular death, MI or ischemic stroke, all-cause death, cardiac death, MI, ischemic stroke, stent thrombosis, TIMI minor or major; BARC 2,3 or 5 bleeding	3 months DAPT followed by ticagreglor monotherapy	12 months
**Twilight DM CKD **[15]	Death, MI or stroke, all-cause death, MI or ST, BARC 2, 3 or 5 bleeding	3 months DAPT followed by ticagrelor monotherapy	12 months
**Tico **[16]	All-cause mortality, cardiac death, MI, stent thrombosis, ischemic stroke, TVR, any revascularization, TIMI minor and TIMI major bleeding, fatal bleeding, BARC 3c	3 months DAPT followed by ticagrelor monotherapy	12 months

*Abbreviations*: *MACE* Major adverse cardiac events, *MI* Myocardial infarction, *TVR* Target vessel revascularization, *POCE* Patient-oriented composite endpoint, *NACE* Net adverse clinical events, *TIMI* Thrombolysis in myocardial infarction, *DAPT* Dual antiplatelet therapy, *BARC* Bleeding defined according to the academic research consortium

The endpoints which were assessed in this study included:

- All-cause mortality;
- Cardiovascular death;
- Myocardial infarction (MI);
- Target vessel revascularization (TVR);
- Stroke;
- Major adverse cardiovascular events (MACEs) including a combination of death, MI and/or stroke/revascularization;
- Stent thrombosis including definitive and probable stent thrombosis;
- Thrombolysis in myocardial infarction (TIMI) defined major bleeding [17];
- TIMI defined minor bleeding [17].

TIMI bleeding is mainly based on laboratory parameters (fall in hematocrit or hemoglobin levels) developed in patients with ST-elevation MI receiving thrombolytic therapy. TIMI minor bleeding was considered when blood loss with ≥ 3 g decrease in the hemoglobin level or ≥ 10% decrease in the hematocrit level was observed whereas TIMI major bleeding was considered when the blood loss was above 5 g decrease in hemoglobin level or above 15% decrease in the hematocrit level.

(j) Bleeding defined by the academic research consortium (BARC) [18].

### Data extraction and quality assessment

The authors Chen Ning, Fang Ling, Deyi Liu and Zhang Zhi independently extracted data from the selected original studies derived from randomized trials. Data that were extracted included the total number of participants with versus without DM who were assigned to ticagrelor monotherapy after a short course of DAPT with ticagrelor and aspirin following PCI, the outcomes which were reported and the total number of events associated with each outcome, the baseline characteristics, the duration of follow-up, the duration of DAPT, and several other information were extracted.

Any disagreement that occurred during the data extraction process was carefully discussed among the authors and a consensus was reached.

Recommendations from the Cochrane collaboration were considered when assessing the methodological quality of the studies [19] whereby grades were allocated to represent low (Grade A), moderate (Grade B) or high risk (Grade C) of bias.

### The statistical analysis of this study

Revman 5.4 software was used to conduct this meta-analysis. Risk ratio (RR) and 95% confidence intervals (CI) were used to represent the results which were generated. Heterogeneity was assessed by the Q statistic test whereby a *P* value less or equal to 0.05 was considered statistically significant. Heterogeneity was also assessed by the I^2^ statistic test whereby a lower I^2^ value represented a low heterogeneity and a higher I^2^ value represented a higher heterogeneity.

When the value of I^2^ was less than 50%, a fixed effect statistical model was used for analysis, whereas when I^2^ value was greater than 50%, a random effect statistical model was used.

Sensitivity analysis was also carried out to rule out the impact of any dominant study on the results. Publication bias was visually assessed through generated funnel plots. The main graphical method for identifying publication bias is the use of funnel plots. A funnel plot is a plot of effect size against sample size or some other indicator of the precision of the estimate. An Egger’s test or Begg test could not be considered since our analysis included less than 10 studies making those tests insignificant.

### Ethics approval and consent to participate

Ethical approval and consent to participate were not applicable for this systematic review and meta-analysis.

## Results

### Search outcomes

The Preferred reporting items in systematic reviews and meta-analyses (PRISMA) reporting guideline was followed in this analysis [20]. Our search resulted in a total number of 8620 publications [2432 from MEDLINE, 1745 from EMBASE, 378 from http://www.ClinicalTrials.gov, 2611 from Web of Science, 856 from Google scholar and 598 from the Cochrane databases]. The authors carefully assessed the titles and abstracts. Irrelevant studies were directly eliminated (7890 articles). Seven hundred and thirty (730) full text articles were assessed for eligibility. Duplicated full-text studies were first of all eliminated (493). Based on the criteria for inclusion and exclusion, studies were further eliminated as followed:

- Meta-analyses and systematic reviews (46);
- Repeated studies derived from the same trials (48);
- Did not involve patients with DM (84);
- Absence of a control group (non-diabetes) for comparison (18);
- Did not involve ticagrelor monotherapy after a short course of DAPT (37);
- Case studies (34).

**Fig. 1:**
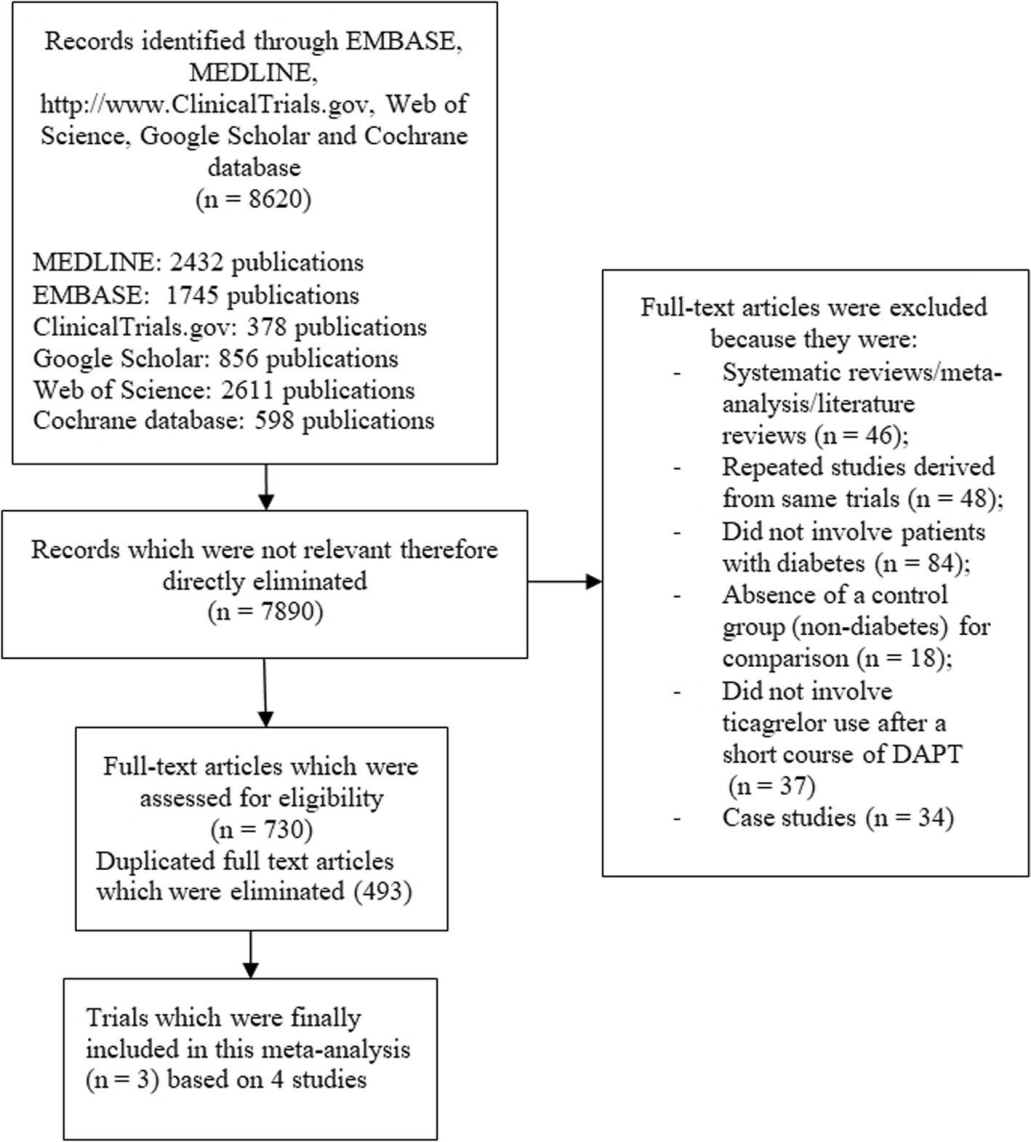
Flow diagram showing the study selection for this meta-analysis

Finally, 4 studies [13–16] derived from 3 randomized trials were included in this analysis (Fig. 1).

### General features and baseline characteristics of the studies

The general features of the trials have been listed in Table 2. A total number of 22,574 participants were included in this analysis (4693 DM and 17,881 without DM). Participants were enrolled from years 2013 to 2019.

**Table 2:** General features of the studies

Studies	Ticagrelor monotherapy in patients with DM	Ticagrelor monotherapy in patients without DM	Participants’ selection	Enrollment period (years)	Year of publication	Bias risk grade
**Global Leaders **[13]	1332	11,351	randomization	2013—2015	2020	B
**Twilight **[14]	-	-	randomization	2015—2017	2020	B
**Twilight DM CKD **[15]	2526	4309	randomization	2015—2019	2022	B
**Tico **[16]	835	2221	randomization	2015 – 2018	2021	B
**Total No of participants (n)**	4693	17,881				

*Abbreviations*: *DM* Diabetes mellitus, *CKD* Chronic kidney disease

Two studies had a follow-up time period of 12 months whereas one study had a follow-up time period of 24 months. In addition, in three studies, the course of DAPT was 3 months followed by ticagrelor monotherapy while in one study, the course of DAPT was for 1 month followed by ticagrelor monotherapy.

The baseline features of the participants have been listed in Table 3. The mean age of the participants with DM ranged from 63.3 to 68.2 years, whereas the mean age of the participants without DM ranged from 60.0 to 67.4 years as shown in Table 3. Male participants varied from 69.3% to 76.6% in the DM group and 71.1% to 81.8% in the group without DM. The mean percentage of patients with previous MI ranged from 23.6% to 71.9%, previous stoke ranged from 2.80% to 7.40% and the mean body mass index varied from 24.9 to 30.1 kg/m^2^ as shown in Table 3. The mean percentages of participants with hypertension, dyslipidemia and those who were smokers have been listed in Table 3.

**Table 3:** Baseline features of the studies

Studies	Age (years)	Males (%)	HBP (%)	Smoker (%)	DL (%)	Previous MI (%)	HbA1c (%)	Previous stroke (%)	BMI kg/m^2^
	**DM/NDM**	**DM/NDM**	**DM/NDM**	**DM/NDM**	**DM/NDM**	**DM/NDM**	**DM/NDM**	**DM/NDM**	**DM/NDM**
**Global Leaders **[13]	68.2/67.3	69.3/71.1	88.7/75.7	17.3/22.7	75.8/65.2	28.8/23.6	-	4.55/2.80	29.8/27.8
**Twilight DM CKD **[15]	65.8/67.4	73.1/73.3	85.0/72.3	15.7/20.3	71.7/60.3	30.1/27.4	-	-	30.1/27.8
**Tico **[16]	63.3/60.0	73.3/81.8	68.3/43.7	31.9/39.4	64.1/59.0	64.0/71.9	7.70/5.90	7.40/2.90	25.1/24.9

*Abbreviation*: *DM* Diabetes mellitus, *NDM* Non-diabetes mellitus, *HBP* High blood pressure, *DL* Dyslipidemia, *CKD* Chronic kidney disease, *MI* Myocardial infarction, *HbA1c* glycated hemoglobin, *BMI* Body mass index

### Main results of the analysis

When analyzing for MACEs subgroup, events occurred in a total number of 265 DM participants out of 4668 patients with DM and 669 participants out of 17833 patients without DM and when analyzing for all-cause mortality, events occurred in a total number of 124 DM participants out of 4251 participants with DM and 336 participants out of 16721 participants without DM. When assessing for cardiovascular death, events occurred in a total number of 20 DM participants out of 1726 patients with DM and 13 participants out of 3325 patients without DM. For stroke, the values were 29 participants with DM out of 3058 patients with DM and 105 participants out of 14676 participants without DM.

**Fig. 2:**
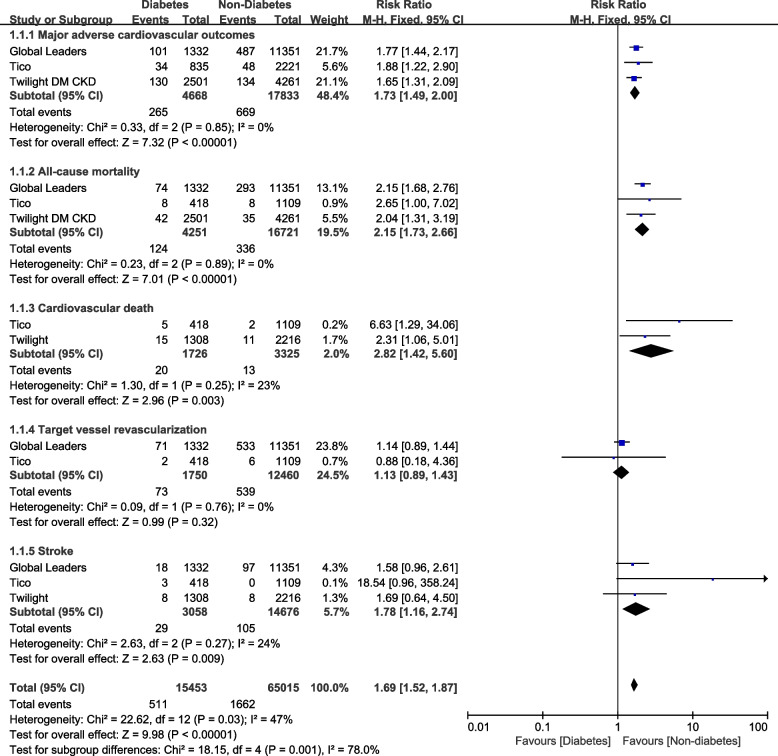
Adverse cardiovascular outcomes with Ticagrelor monotherapy after a short course of dual antiplatelet therapy (DAPT) with Ticagrelor plus aspirin following percutaneous coronary intervention (PCI) in patients with versus without diabetes mellitus

**Fig. 3:**
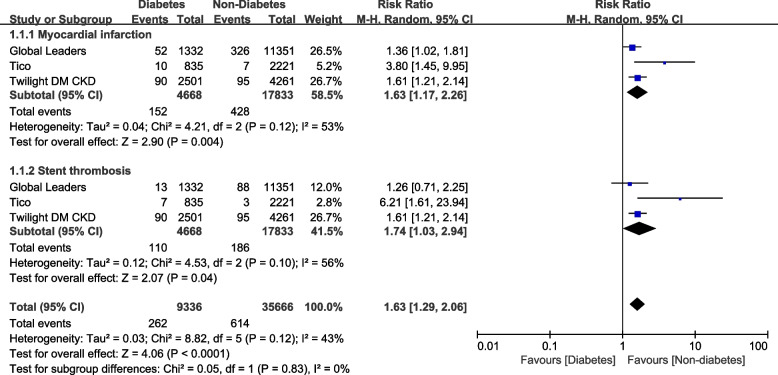
Myocardial infarction and stent thrombosis with Ticagrelor monotherapy after a short course of DAPT with Ticagrelor plus aspirin following PCI in patients with versus without diabetes mellitus

Results of this analysis showed that in patients with ticagrelor monotherapy after a short course of DAPT, DM was associated with significantly higher risks of MACEs (RR: 1.73, 95% CI: 1.49 – 2.00; *P* = 0.00001; I^2^ = 0%), all-cause mortality (RR: 2.15, 95% CI: 1.73 – 2.66; *P* = 0.00001, I^2^ = 0%), cardiac death (RR: 2.82, 95% CI: 1.42 – 5.60; *P* = 0.003, I^2^ = 23%) and stroke (RR: 1.78, 95% CI: 1.16 – 2.74; *P* = 0.009, I^2^ = 24%) when compared to patients without DM as shown in Fig. 2. However, TVR was not significantly different (RR: 1.13, 95% CI: 0.89 – 1.43; *P* = 0.32, I^2^ = 0%). MI (RR: 1.63, 95% CI: 1.17 – 2.26; *P* = 0.004, I^2^ = 53%) and stent thrombosis (RR: 1.74, 95% CI: 1.03 – 2.94; *P* = 0.04, I^2^ = 56%) were also significantly higher in the DM group as shown in Fig. 3.

When assessing for the bleeding outcomes, events occurred in 81 participants out of 2154 patients with DM and 126 patients out of 4457 participants without DM for TIMI minor bleeding and 88 DM patients out of 2154 participants with DM versus 123 participants out of 4457 participants without DM were observed for TIMI defined major bleeding. And for BARC 2,3 or 5, events occurred in 256 DM participants out of 3858 participants with DM versus 969 patients out of 15660 participants without DM.

**Fig. 4:**
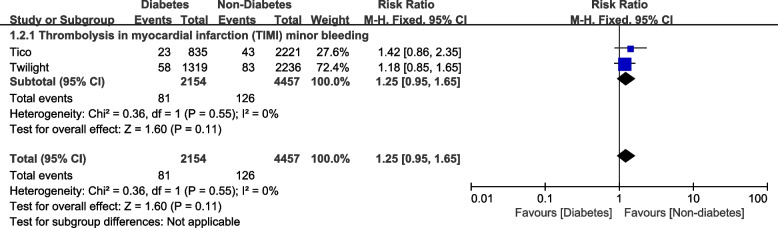
Thrombolysis in myocardial infarction (TIMI) defined minor bleeding observed with Ticagrelor monotherapy after a short course of DAPT with Ticagrelor plus aspirin following PCI in patients with versus without diabetes mellitus

**Fig. 5:**
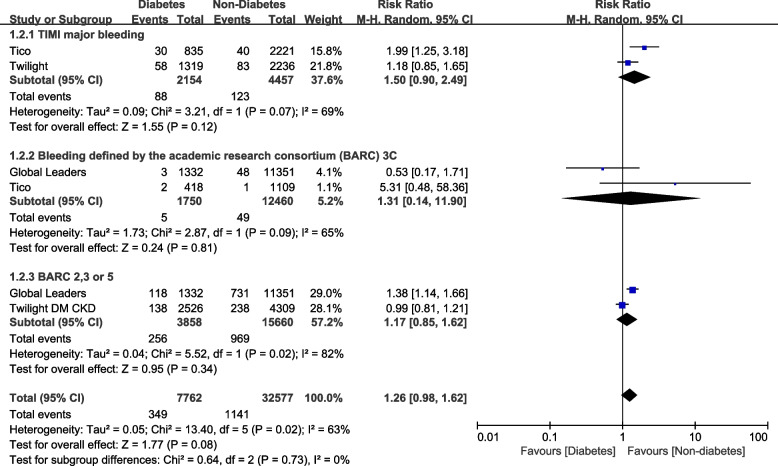
TIMI defined major bleeding and bleeding defined by the academic research consortium (BARC) observed with Ticagrelor monotherapy after a short course of DAPT with Ticagrelor plus aspirin following PCI in patients with versus without diabetes mellitus

When the bleeding outcomes were compared, TIMI defined minor (RR: 1.25, 95% CI: 0.95 – 1.65; *P* = 0.11, I^2^ = 0%) was not significantly different as shown in Fig. 4. In addition, TIMI defined major bleeding (RR: 1.50, 95% CI: 0.90 – 2.49; *P* = 0.12, I^2^ = 69%), BARC 3c bleeding (RR: 1.31, 95% CI: 0.14 – 11.90; *P* = 0.81, I^2^ = 65%) and BARC 2, 3 or 5 (RR: 1.17, 95% CI: 0.85 – 1.62; *P* = 0.34, I^2^ = 82%) were not significantly different among patients with versus without DM as shown in Fig. 5.

Consistent results were shown throughout during sensitivity analysis. For a better clarity, using a method of exclusion, each study was excluded one by one and a new analysis was therefore carried out each time and the results which were obtained were carefully compared with the main results of this analysis to observe for any significant change. However, no significant change was observed when sensitivity analysis was carried out indicating that the results were not influenced by data from any particular study. For example, when study Global Leaders was excluded from the analysis and a new analysis was carried out, the significances of MACEs (RR: 1.70, 95% CI: 1.38 – 2.09; *P* = 0.00001), all-cause mortality (RR: 2.13, 95% CI: 1.42 – 3.20; *P* = 0.0002), MI (RR: 1.73, 95% CI: 1.32 – 2.26; *P* = 0.0001, TVR (RR: 0.88, 95% CI: 0.18 – 4.36, *P* = 0.88), stroke (RR: 2.44, 95% CI: 1.03 – 5.75, *P* = 0.04) and stent thrombosis (RR: 1.72, 95% CI: 1.31 – 2.26; *P* = 0.0001) were similar to the main results of this analysis, there was no significant difference in the result findings. This could explain the consistence of this sensitivity analysis.

**Fig. 6:**
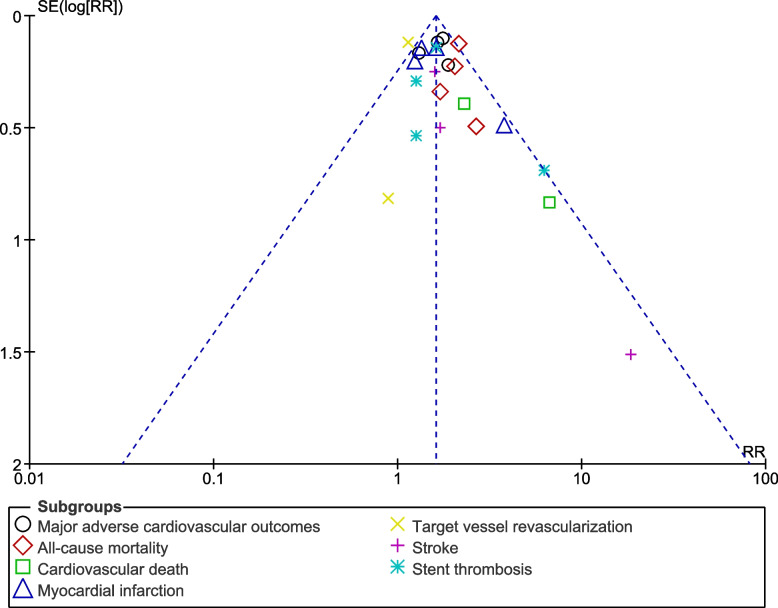
Funnel plot showing publication bias (A)

**Fig. 7:**
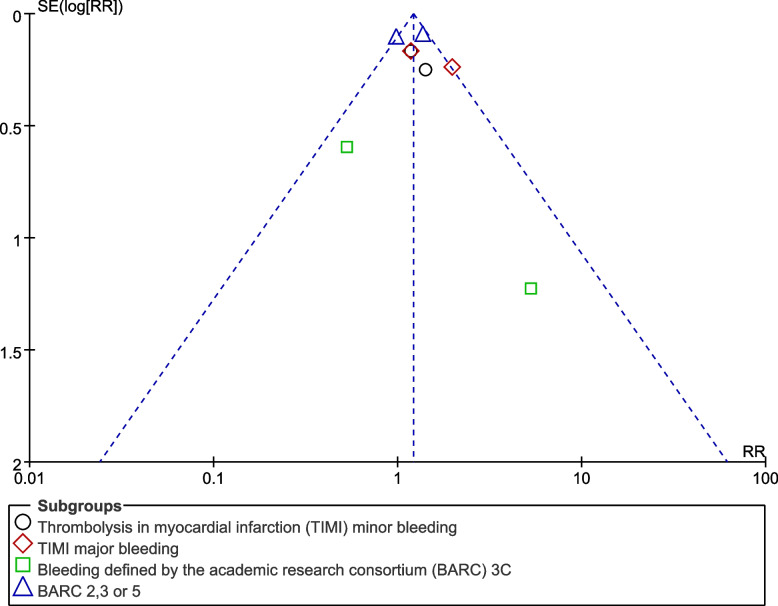
Funnel plot showing publication bias (B)

Publication bias was represented by Figs. 6 and 7 whereby the funnel plots which were generated were visually analyzed based on their symmetrical aspect. There was only little evidence of publication bias across the studies which were considered during data analysis. The funnel plots which were generated were almost fully symmetrical and this could explain the low evidence of publication bias.

## Discussion

In this analysis, we aimed to compare the clinical outcomes observed in patients with versus without DM who were assigned to ticagrelor monotherapy after a short course of DAPT with ticagrelor and aspirin following PCI.

The current results showed that MACEs, MI, all-cause mortality, cardiac death, stroke and stent thrombosis were significantly higher in the DM group. TIMI defined major and minor bleedings as well as BARC defined bleeding were not significantly different.

DM has always been an independent risk factor for adverse cardiovascular outcomes following PCI. Previous studies have shown DM to be an independent risk factor for the development of adverse cardiovascular outcomes whether with or without insulin therapy [21], whether with different coronary stents [22] or with prolonged duration of DAPT [23]. Studies have also shown that the longer the duration of DM, the higher the major adverse cardiovascular events observed after PCI. Benjamin et al. aimed to assess the association between DM duration and MACEs post-PCI [24]. The study comprised of a total of 302 cases of DM patients who underwent PCI with drug eluting stents. There was three different categories of patients including those less than 5 years of disease duration, 5 to 10 years of disease duration and those greater than 10 years of DM duration. The authors demonstrated that the greater the duration of DM, the higher the adverse cardiovascular events.

Another study which was a retrospective study with patients with coronary artery disease undergoing PCI [25], enrolled from 2009 to 2018, patients with DM had a higher disease risk profile, especially those on insulin therapy. With a longer follow-up time period, patients with DM presented a higher risk of MACEs following PCI and this risk was more intense in DM patients on insulin therapy. However, the study did not show any difference in MACEs between patients with non-insulin treated DM and those without DM. A meta-analysis including a total number of 139,774 participants showed that when compared to patients without DM, patients with DM had a significantly higher risk of in-hospital, short term, and long-term adverse clinical outcomes following PCI [26].

In another recent meta-analysis the authors aimed to show the long term ticagrelor monotherapy for the treatment of patients with type 2 DM following PCI [27]. A total number of 8621 patients were included. The authors showed that long-term ticagrelor monotherapy after a short course of DAPT use resulted in better outcomes significantly decreasing the risk of MACEs and all-cause mortality, and significantly decreasing the risk of TIMI defined minor and major bleedings when compared to DAPT use. However, in our current analysis, we compared ticagrelor monotherapy in patients with and without DM in contrast to the other analysis which compared ticagrelor monotherapy versus DAPT in patients with DM.

A meta-analysis which compared the efficacy and safety of P2Y12 inhibitor monotherapy and DAPT in patients with and without DM undergoing PCI showed that this monotherapy did not increase the risk of MACEs in patients with DM [28]. In fact, this monotherapy was associated with a lower risk of MACEs in the DM population. The authors also demonstrated that even though bleeding events were reduced, this reduction in bleeding events was much more significant in patients without DM when compared to patients with DM. Our current meta-analysis compared ticagrelor monotherapy in patients with and without DM, in contrast to this recently discussed meta-analysis which compared ticagrelor monotherapy versus DAPT in patients with and without DM following PCI.

TIMI defined minor and major bleedings were not significantly different in this current study. Previous studies have shown DM to be associated with a higher risk of thrombosis following PCI even with the use of DAPT involving aspirin and clopidogrel. This higher risk of thrombosis was due to platelet hyperactivity in the DM population [29]. In addition, several patients suffered from aspirin and clopidogrel hyporesponsiveness [30] which could also contribute to this high risk of thrombosis. Previously published studies warranted more potent anti-platelet agents in patients with DM [31].

Our current analysis showed ticagrelor monotherapy followed by a short course of DAPT with ticagrelor and aspirin not to be associated with significantly higher bleeding risks. The THEMIS-PCI trial [32] showed that DAPT with ticagrelor and aspirin in patients with DM could result in increased major bleeding and therefore the authors suggested the use of this ticagrelor based DAPT in DM patients who have tolerated antiplatelet therapy, high ischemic risk with low bleeding risk. Hence, ticagrelor monotherapy could well be tolerated in DM patients without paying significant attention to high or low ischemic or bleeding risk. In addition, a European economic evaluation of the cost-effectiveness of ticagrelor in DM patients with coronary artery disease based on THEMIS trial results showed ticagrelor plus aspirin in comparison to aspirin alone could be cost-effective in some European countries in patients with DM and coronary artery disease and the authors also stated that ticagrelor might be cost effective across European countries in patients with a history of PCI [33].

### Limitations

This study also has limitations. First of all, the total number of original studies which have been used in this analysis was less compared to other meta-analyses. Including a total of only 4 studies based on 3 trials could act as a limitation in this analysis. In addition, two studies reported DAPT use with ticagrelor and aspirin for 3 months prior to ticagrelor monotherapy whereas one study reported DAPT use for only one month. This could have had an impact on the outcomes which were obtained. Also, the cardiac drugs which were used by the patients were completely ignored in this analysis. Moreover, another limitation could be the fact that the severity of disease was not similar in all the original studies. Two studies also involved patients with chronic kidney diseases who are at higher risks of complications .

## Conclusions

In patients who were treated with ticagrelor monotherapy after a short course of DAPT with ticagrelor and aspirin, DM was an independent risk factor for the significantly increased adverse cardiovascular outcomes. However, TIMI and BARC defined bleeding events were not significantly different in patients with versus without DM.

## Data Availability

Data which have been used in this study can freely be accessed and are included in the original published articles. References of the original papers involving the data source which have been used in this paper have been listed in the main text of this current manuscript. All data are publicly available in electronic databases.
